# Guillain-Barre syndrome: A possibility in a spinal cord injured patient

**DOI:** 10.4103/0019-5413.33690

**Published:** 2007

**Authors:** Yogendrasinh Jagatsinh

**Affiliations:** Yorkshire Regional Spinal Injury Centre, Pinderfields General Hospital, Wakefield, UK

**Keywords:** Guillain-Barre syndrome, progressive paralysis, syringomyelia, spinal cord injury

## Abstract

A 28-year-old male had paraplegia as a result of fracture dislocation of T12/L1 six years ago. He was functioning independently until four weeks ago, when he started complaining of trunkal paraesthesia which later progressed to include the upper extremities. The initial diagnosis was that of posttraumatic syringomyelia (PTS). While awaiting the MRI scan he developed weakness of upper limbs. The weakness restricted his self-care activities including transfers. The MRI did not show any evidence of syringomyelia. Neurological consultation and assessment yielded provisional diagnosis of Guillain-Barre syndrome (GBS). The patient was treated with immunoglobulins and regained 90% of his previous neurological status. This case is reported to raise awareness among clinicians to include the possibility of the GBS in the differential diagnosis of progressive neurological loss on top of existing neurological deficiency in spinal cord injured patients.

As spinal surgeons we all are aware of posttraumatic syringomyelia, tethered cord syndrome and subacute posttraumatic ascending myelopathy to cause neurological deterioration in old spinal cord injury. Here we present a case where our patient had medical condition, diagnosed as Guillain-Barre syndrome (GBS) leading to neurological deterioration.

## CASE REPORT

A 28-year-old gentleman sustained a fracture dislocation of T12/L1 with paraplegia below T10 (UMN lesion), American Spinal Injury Association (ASIA) scale C, six years ago following a road traffic accident. He was managing quite well until four weeks ago. He initially started developing paraesthesia on the trunk. On thorough examination no alteration in motor power was found but he had scattered loss of sensation higher up in the T6 to T12 region. An MRI scan was requested considering syringomyelia as diagnosis on outpatient basis. After four days he presented to us with excruciating pain, severe weakness and altered sensation in both upper limbs. He was immediately admitted and on examination showed tender muscles with marked decrease in power, wasting of the distal muscles and a glove and stocking type of sensory impairment in both upper limbs up to the shoulder and he was areflexic. His dorsal column sensations were also moderately affected whilst all deep tendon reflexes were found to be normal. An immediate MRI was arranged which showed normal appearance in his spinal cord and no evidence of syrinx. He was referred to neurologists and with investigations like full blood count, thyroid function tests, protein electrophoresis immunoglobulin, viral markers, toxic heavy metal screening, porphyria screen, auto-antibodies, EBV viral capsate and nerve conduction studies (which showed extensive peripheral axonal damage), GBS was considered. He was kept under strict monitoring of vital capacity and cranial nerve affections. He was not subjected to CSF analysis due to extensive metal work in his spine in the thoracolumbar region. He was treated with IV immunoglobulin for five days and after a week his condition was better clinically and was discharged home. After six months' follow-up in outpatient he regained 90% of his previous neurological status and is still continuously improving.

## DISCUSSION

The GBS was described in 1916 by three French neurologists Georges Guillain, Jean-Alexandre Barre and Andre Strohl in two soldiers with acute areflexic paralysis followed by recovery.^1^ It is an eponym for a heterogeneous group of immune-mediated peripheral neuropathies. The Guillain-Barre syndrome generally manifests as a symmetric motor paralysis with or without sensory and autonomic disturbances.^2^ Cranial nerve involvement may affect airway maintenance, facial muscles, eye movements and swallowing. Patients should be hospitalized for observation.^2^ Pain, another common feature of GBS, is seen in approximately one half of all patients and is sometimes described as severe, occurring with even the slightest of movements. Pain is more severe in the shoulder girdle, back and posterior thighs.^3^,^4^ Functional recovery can be expected within six to 12 months. However, some patients have persistent minor weakness, areflexia and paraesthesia. Approximately 7-15% of patients have permanent neurological sequelae.

Posttraumatic syringomyelia (PTS), first described by Bastian in 1867, is a common cause of ascending neurological deterioration in spinal cord injury (SCI). Posttraumatic syringomyelia is characterized clinically by the often insidious progression of pain and loss of sensorimotor function that may manifest after years after traumatic SCI. If left untreated, PTS can result in loss of function, chronic pain, respiratory failure or death. Other causes to be considered are spinal cord tumor, spinal cord infarct, epidural abscess or hematoma, tethered cord syndrome, progressive noncystic myelopathy, spinal instability. Vertebral trauma is the second most common cause of syringomyelia.^5^

Early surgical treatment is highly recommended before the establishment of gross neurological deficits occurs. Treatment is urged in case of clinical deterioration or when the follow-up MRI studies show increase in size and extension of syringomyelic cavity.^5^ The first step in the surgical treatment is a precise diagnosis of its etiology to direct the treatment to the underlying cause.

Pain, increased spasticity and hyperhidrosis are typical differentiating features of syringomyelia. These can be aggravated by the valsalva maneuver. These are typically absent in GBS. Progressive neurological weakness and wasting are late in syringomyelia. Lack of deep tendon reflexes is a hallmark of GBS while it is exaggerated in syringomyelia. Magnetic Resonance Imaging is the investigation of choice for syringomyelia.

We present a case of GBS where most of the spinal cord injury physicians would make first diagnosis as post traumatic syringomyelia when long standing paraplegic presents with neural deterioration.

The GBS, can affect spinal cord injured patients and can lead to life-threatening complications. The incidence of typical GBS has reported to be relatively uniform between 0.6 and 4 cases per 100,000 per year throughout the world. Men are about 1.5 times more likely to be affected than women. Most cases are sporadic, but small clusters have been associated with outbreaks of bacterial enteritis caused by contaminated water and summer epidemics occur in northern China, probably caused by *Campylobacter jejuni* infection.^1^ The diagnosis of GBS itself is usually not difficult for the neurologist, but can be challenging for the doctor of first contact who may not have seen a case since medical school. Established diagnostic criteria exist and have stood the test of time. The differential diagnosis is wide and depends first on the clinician recognizing that the problem is an acute peripheral neuropathy and not a brainstem, spinal cord or conus lesion. Supportive care remains the cornerstone of therapy. If patients advance past the acute phase of illness, most will recover function. However, the neuropathy can advance so rapidly that endotracheal intubation and mechanical ventilation may be necessary within 24h of symptom onset; for this reason, all patients who are suspected to have GBS should be admitted to the hospital for close observation for respiratory compromise, cranial nerve dysfunction and autonomic instability. To the author's best knowledge there are no published case reports or statistics or studies on GBS in SCI patients.

We believe that all SCI physicians should keep this diagnosis in mind while dealing with similar patients. We believe that all our patients complaining of acute change in neurology should be admitted and investigated to rule out causes other than syringomyelia.
